# Letter from Dr. B. T. Whitney

**Published:** 1849-01

**Authors:** 


### LETTER FROM DR. B. T. WHITNEY.

*Messrs. Editors:*

From the love I bear our profession, I feel bound to expose and reprove quackery, particularly when hid behind the glitter of some gaudy tinsel, or commanding respect from its boldness, or the high position of its *illustrious* venders. I have, therefore, thought proper to make the following reflections:

On being introduced to a Dr. ——, of Kentucky, a short time since, he very politely handed me his *pamphlet*, from which I make the following extracts: "He would here remark, that he has laid aside the instrument for extracting the nerve, and uses a *chemical* preparation which causes no pain, and tends to preserve the teeth." (?) "A lady of my acquaintance informs me that a friend of hers was held while a *Dentist* (?) thrust a red-hot wire into the cavity of the tooth, thereby causing the most excruciating pain, and this, forsooth, to destroy the nerve of a tooth"!!

He brings to his support the following: "A gentleman from Boston called on me last summer to consult with me about the health of his teeth. I found but one in the least defective, and that had been plugged thirty years since by a distinguished dentist of that city, Dr. Flagg. The pulp had been entirely destroyed at that time."

His pamphlet is also well filled with certificates from doctors, &c., of his skill in nerve killing—forming a beautiful chariot in which to ride into practice. The following are extracts of but two of the number, and signed by physicians: "As it respects your practice here, so far as my observation goes, I was well pleased. * * * My impression is, that your plan of destroying the nerve of a tooth, before plugging, is good in practice." "I have observed his work, particularly in extracting, killing the nerve, and plugging of teeth, and I think him very skilfull."

Certificates like these, published to the world as coming from practising physicians, are bound to exert an influence over most people, particularly if the operator is doing an itinerant business, so that his works do not follow him. They teach us two things: that the physician and clergyman are not competent judges of skill in the dental art; and that the merest empyric can procure an abundance of such capital.

On a jaunt into Kentucky a short time since, I saw posted up the bills of a "Dentist and Daguerreotypist," advertising in glowing style, that after great efforts *he* had *discovered a chemical* by which he could destroy the nerves of teeth without the *least pain*; and his "*skill in plugging would save the teeth thus treated for life.*" Wishing to add what I could to my little stock of knowledge, I sought this wonderful young man's acquaintance, and found that he emanated from an *extensive* office in Boston; and that, with his uncle of that city, he had made this wonderful discovery—of course, out of pure magnanimity for suffering humanity;—that during the first month after they *advertised* it, he destroyed twelve hundred nerves, at fifty cents each; (?) that it was the only thing that ever should be used; that teeth never *rotted* after it; and that it remained a profound secret!!

I asked him why he did not give to the world, or the profession at least, a *discovery* that *promised* so much. He replied: "as they had spent much time in *testing chemicals*, they chose to reap the benefit of their own labors."

But, instead of offering to *buy* his recipe or *chemical*, I very ungenerously denounced his *discovery* as worthless; the practice of nerve killing, as unprofessional; his pretensions, as deceiving the best interests of his patients, though in an itinerant business he might pick up some money;—that I deprecated every secret nostrum or patented improvement in Dentistry, as an insult to the profession, and an abuse to the public. Our motto is, "onward;" and I hold that every man should give his improvements for the advancement and elevation of the profession.

I have alluded to these two cases by way of illustration, not as of rare occurrence; for, unfortunately, almost every town and hamlet is infested. It may be laid down as an established axiom, that men who *travel* in such a chariot, are not only wanting in real worth as men, but are destitute of professional knowledge. Unfortunately for the victim, the world judges at first more from the *outward show*, than the *inward worth ;* and to this credulity is the empiric mainly indebted for his support.

Yours truly, B. T. WHITNEY.

CLARKSVILLE, Tenn., Sept. 23, 1848.

